# Patterns of breastfeeding practices among infants and young children in Abu Dhabi, United Arab Emirates

**DOI:** 10.1186/s13006-018-0192-7

**Published:** 2018-11-16

**Authors:** Zainab Taha, Malin Garemo, Joy Nanda

**Affiliations:** 1 Department of Health Sciences, Zayed University, PO Box 144534, Abu Dhabi, United Arab Emirates; 2 The John Hopkins Medical Institutions, Baltimore, MD USA

**Keywords:** Exclusive breastfeeding, Complementary feeding, Infant nutrition, Toddler, Abu Dhabi, United Arab Emirates

## Abstract

**Background:**

Despite the ongoing efforts to improve infant feeding practices, low rates of breastfeeding and early introduction of complementary feeding have been reported in many countries. Systematic documentation of breastfeeding practices in the United Arab Emirates (UAE) is needed in order to directing successful strategies. The aim of this study was to evaluate breastfeeding practices among mothers in Abu Dhabi, UAE, using the World Health Organization (WHO) infant and young child feeding indicators.

**Methods:**

In this cross-sectional study, mothers of children below the age of two were recruited from the community and health centers located in different areas in Abu Dhabi. Following informed consent, a structured questionnaire including WHO-indicators was used for in-person interviews on sociodemographics and breastfeeding. Exclusive breastfeeding (EBF) was calculated as the percentage of babies 0 – < 6 months of age who had been exclusively breastfed in the last 24 h.

**Results:**

A total of 1822 mothers participated in the study; 95.6% (1741/1822) of mothers initiated breastfeeding and 59.8% (1089/1822) initiated breastfeeding within the first hour. Exclusive breastfeeding among infants 0–6 months was 44.3% (362/818). Although the median duration of “any breastfeeding” was 12 months (95% CI 11.2, 12.7), the median duration of EBF was 3 months (95% CI 2.8, 3.3). Most of the children (894/1004, 89%) aged 6 months and above were receiving complementary feeding, but 21.7% (218/1004) of them had had an early introduction of complementary feeding, i.e. before 6 months of age. Using “the WHO infant and young child feeding indicators” as standard for comparative evaluation, breastfeeding initiation was rated “good”, the proportion of children being exclusively breastfed until 6 months was rated “fair” and the duration of EBF was considered “poor”.

**Conclusions:**

According to the WHO infant feeding indicators the breastfeeding practices were suboptimal in several aspects with a low proportion of children being exclusively breastfed, short breastfeeding duration and early introduction of complementary feeding, despite high socioeconomic status. These findings suggest that there is a need to understand potential barriers towards breastfeeding in order to develop appropriate strategies to promote and support breastfeeding in Abu Dhabi.

## Background

The feeding practices from birth to 24 months are of utmost importance to meet the evolving nutritional requirements of infants and young children. Breastfeeding has been proven to reduce the risk of childhood diseases and malnutrition [1]. Moreover, breastfeeding is also a contributing factor in preventing non-communicable diseases such as obesity, diabetes, and cardiovascular diseases in the later stages of life [2–4]. There is also evidence that breastfeeding decreases the incidence and/or severity of a wide range of infectious diseases [5] and is known to have a positive impact on the immune system, cognitive, emotional and social development [6].

Appropriate nutrition during infancy and childhood is a prerequisite for optimal child health and development [7]. Consequently, the global strategy for infant and young child feeding recommends that breastfeeding should be initiated within the first hour after birth, and breast milk exclusively, should be used to feed infants during the first 6 months of life [8]. From 6 months of age, breastmilk does not adequately supply the infants’ daily requirements of certain nutrients like iron and vitamin D, and therefore complementary feeding is needed [9]. Hence, in addition to the recommendation of exclusive breastfeeding from birth to 6 months of age, the WHO also recommends timely initiation of safe, nutritionally-adequate and age-appropriate complementary feeding at 6 months with continued breastfeeding up to 2 years of age or above [10]. It has been shown that nutritional adequacy and proper timing of complementary feeding are crucial to the prevention of infant morbidity and mortality, including malnutrition and overweightness [11, 12]. Based on the recommendations, the WHO has designed a tool where a set of indicators related to infant feeding practices are helping users, like governments, to determine strengths and weaknesses and potential need for improvements related to exististing practices, policies and procedures [13] .

Although the UAE has adopted the WHO and United Nations Children’s Fund (UNICEF) recommendations for feeding infants and young children, results of the United Arab Emirates (UAE) Family Health Survey from 2000, the only national survey that has been conducted since the country adopted the WHO recommendations, show that only 34% of infants were exclusively breastfed for up to 4 months of age [14]. More recent studies have looked at children from parts of the UAE, used smaller sample sizes compared to the Family Health Survey, and have mainly investigated practices of UAE nationals [15–17].

Traditionally, as a Muslim country, breastfeeding was the normal means of feeding infants and young children in the UAE [18]. Following the rapid economic transition of the UAE, it has grown into a modern, multinational and urbanized society, with several societial changes that may influence mothers willingness, ability and desire to breastfeed their babies. More females are now getting tertiary education, and consequent to the economic transition, female employment has increased from 29.2% in 1990 to 40.9% in 2017, a change that may have impacted the feeding practices of infants and young children by their mothers [19]. Investments in healthcare facilities and improved living conditions have resulted in a steep decline in infectious diseases whereas the nutrition transition and a sedentary lifestyle has lead to an increase in non-communicable diseases [20–22]. A high prevalence of overweightness and obesity is noticeable among children but also underweightness and micronutrient deficiencies have been documented in children [23, 24]. According to the Ministry of Health, nutrition-related chronic diseases are now major causes of morbidity and mortality in the UAE [25]. The Emirates of Abu Dhabi recently released a report on how to combat childhood obesity in the Emirates, and one of the main objective is to increase the breastfeeding prevalence [26, 27]. Presently, there is very little documentation on the current feeding practices among infants and young children in Abu Dhabi. It is therefore critical to determine if the current nutritional practices among infants in Abu Dhabi are optimal, or require more effective guidelines and interventions in order to achieve the goals of the UAE vision 2021 [28]. The aim of this study was to evaluate breastfeeding practices among mothers in Abu Dhabi, UAE, and to compare them to the recommendations of the WHO. Comparative analysis was not part of the objective, but are planned to be reported in future papers.

## Methods

### Participants and data collection

The Abu Dhabi Emirate, one of seven emirates (states) in the UAE, consists of three regions, where the Abu Dhabi capital district is the largest in terms of area and population density. In 2016, 40,505 (19,780 girls and 20,725 boys) children aged 0–4 years were residing in the capital district of Abu Dhabi [29]. There are eleven governmental maternal and child health centers in the urban, suburban and rural parts of Abu Dhabi capital district, providing health services for both Emirati and non-Emirati families [29]. As health insurance is mandatory in the UAE, both groups have access to healthcare.

**Fig. 1:**
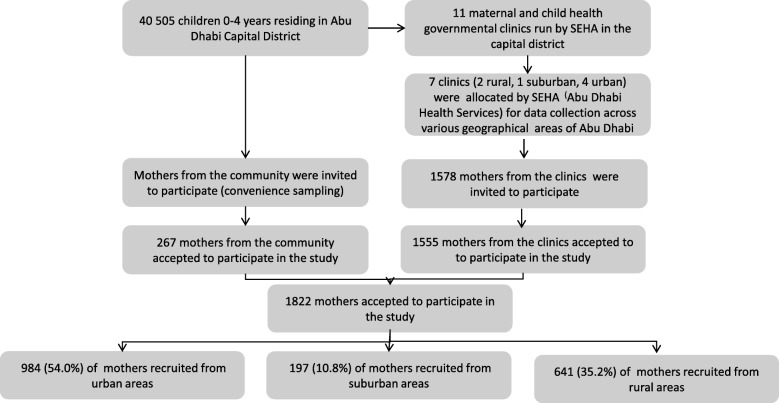
Schematic diagram of the overall recruitment of study participants

In this cross-sectional study the sample was recruited from the community (mainly university students) and from seven of the maternal and child health centers, spread across different geographical areas. Figure 1 displays the schematic way of recruiting participants to the study. Trained bilingual (Arabic and English) research assistants (RAs) interviewed mothers both at the healthcare centers and in the community at different times and days during the period of March–September 2017. All eligible mothers visiting the health centers during these timings were invited to participate in the study. Oral and written information was given to participants by the RAs about the study. One thousand eight hundred and twenty two consenting mothers from both the healthcare centers and the community who met the inclusion criteria of having at least one healthy child under 2 years of age were interviewed by an RA using a structured questionnaire.

### Study instrument

The questionnaire consisted of 57 main questions with several sub items. It was initially developed in English and then translated into Arabic using a cross-translation technique whereby a native Arabic speaker translated the English document into Arabic, followed by a second native Arabic speaker (blind to the original document) retranslating the document back into English. Any translation errors identified were reviewed by the research team and modified to minimize semantic errors. A pilot study was conducted where face validity was utilized by the principal investigator, in order to validate the questionnaire prior to distributing it.

Birthweight and length were provided from the children’s health cards. The questionnaire included questions about family demographic characteristics (e.g. education, age, self-rated financial status, nationality, occupation, parity) and infants information (e.g. birth order, delivery mode). It furthermore included questions on infant’s feeding practices (e.g. initiation of breastfeeding, duration of exclusive breastfeeding, any breastfeeding, formula feeding, and age at which complementary feeding was introduced). The WHO “Infant and Young Child Feeding Tool - A tool for assessing national practices, policies and programmes” was used to evaluate the feeding practices of the participants [13]. The questions were designed to address four of the WHO indicators, initiation of breastfeeding, exclusive breastfeeding under 6 months, duration of breastfeeding, and introduction of complementary feeding. According to the WHO tool, each indicator is rated either as “poor”, “fair”, “good” or “very good” depending on the obtained results. The initiation of breastfeeding was calculated as the percentage of babies who were breastfed within 1 h of birth. Exclusive breastfeeding was calculated as the percentage of babies 0 – < 6 months of age who had been exclusively breastfed in the last 24 h. The duration of breastfeeding was calculated as the median duration in months of breastfeeding of children under 3 years. Complementary feeding was calculated as the percentage of babies aged six through 9 months who received complementary foods in the last 24 h while continuing to be breastfeed. In order to prevent any misunderstandings, the RAs explained each feeding practice to the mother prior to asking questions pertaining the specific variables during the interview.

### Statistical analysis

All analyses were performed using IBM SPSS Statistics Premium V.24.0 (IBM SPSS v24.0 Premium, Chicago), STATA v14.0 SE (StataCorp. 2015. Stata Statistical Software: Release 14. College Station, TX: StataCorp LP), and SAS 9.2 (SAS Institute Inc., SAS 9.1.3 Help and Documentation, Cary, NC: SAS Institute Inc.). Frequency distributions and cross-tabulations generated descriptive statistics to evaluate the sociodemographic characteristics of participating mothers and their infants or toddlers, considered as the index child for the survey. Distribution of initiation, frequency, duration, and termination of breastfeeding, including exclusive breastfeeding, were also examined. Since interviews were conducted at different ages of children up to 2 years of age and a significant number of infants and toddlers had ceased breastfeeding by the time of interview, a Kaplan-Meier survival analysis was conducted to determine the cumulative breastfeeding experience (exclusive or any) of all the children in the study. Additionally, and wherever appropriate, group differences were compared using Chi-square tests for proportions and analysis of variance for mean. Infant feeding practices were evaluated and compared to the WHO indicators [13].

### Definitions

Early initiation of breastfeeding was defined as the proportion of children who latched the breast within 1 h of birth. Exclusive breastfeeding was defined as the infant being fed only breast milk without any other oral intake except medications and vitamins within the last 24 h. Complementary feeding was defined as the introduction of any food apart from breastfeeding or infant formula.

## Results

### Sociodemographic and child characteristics

One thousand eight hundred and twentytwo respondents with a mean ± SD age of 30 ± 5 years were successfully interviewed and participated in the study. The majority of the mothers were married, most parents had a university education and most reported their financial wellbeing as very good to excellent. About one third of the sample was Emirati mothers whereas the others were non- Emirati mothers (Table 1).

**Table 1:** Family demographic and health characteristics

Characteristics	*n* = 1822 (%)
Mother’s age (years)	
17–19	20 (1.1)
20–24	247 (13.6)
25–34	1152 (63.2)
35–51	403 (22.1)
Mother’s nationality	
Emirati	596 (32.7)
Non Emirati-Arab	611 (33.5)
Non Emirati-Non Arab	615 (33.8)
Marital status	
Married	1787 (98.1)
Unmarried	35 (1.9)
Mother’s education^a^	
Below high school	80 (4.4)
High school	339 (18.8)
University	1382 (76.7)
Father’s education^b^	
Below high school	39 (2.1)
High school	205 (11.3)
University	1570 (86.5)
Mother’s employment status	
Employed	722 (39.6)
Not employed	1100 (60.4)
Family financial wellbeing	
Excellent/Very good	1211 (66.5)
Good	487 (26.7)
Fair	106 (5.8)
Poor/Very poor	18 (1.0)

^a^21 missing data, ^b^8 missing data

The child characteristics are shown in Table 2. Forty-five percent of the children were below 6 months old, 27% of the children were 6–12 months old and 28% were 1–2 years old. The mean ± SD age of the children was 8.1 ± 5.9 months and the mean ± SD birthweight was 3074 ± 534 g for all children, with infants being born on term weighing 3127 ± 474 g and preterm infants weighing 2352 ± 727 g. The mode of delivery was cesarean section for approximately one third of the mothers and the remaining children were delivered vaginally (Table 2).

**Table 2:** Child characteristics (*n* = 1822)

Child characteristics	*n* (%)
Child’s age at interview (months)	
< 1	20 (1.1)
1–3	395 (21.7)
4–6	403 (22.1)
7–12	495 (27.2)
13–18	296 (16.2)
19–24	213 (11.7)
Gender^a^	
Female	932 (51.5)
Male	879 (48.5)
Delivery Type^b^	
Vaginal	1252 (68.9)
Cesarean Section	566 (31.1)
Delivery hospital	
Baby Friendly accredited^c^	755 (41.4)
Non Baby Friendly accredited	1067 (58.6)
Gestational Age^d^	
< 37 weeks	128 (7.1)
≥38 weeks	1681 (92.9)
Birth Weight (grams)	
< 37 weeks (mean ± SD)	2351 ± 727
≥38 weeks (mean ± SD)	3127 ± 424

^a^11 missing data

^b^4 missing data

^c^Baby Friendly accreditation according to the WHO and UNICEF standard

^d^13 missing data

### Breastfeeding initiation

In this study, of the 1822 mothers, 1741 (95.6%) initiated breastfeeding. There was a significant difference in initiation rate depending on nationality group. The initiation of breastfeeding among Emirati mothers was 92.6% and the corresponding rates among non-Emirati-Arab mothers and non-Emirati-non-Arab mothers were 95.9 and 98.0%, respectively (*p* < 0.001). Mothers who delivered via cesarean section were less likely to initiate breastfeeding compared to those who delivered vaginally with 8 and 2.9% proportion of mothers not initiating breastfeeding respectively (*p* < 0.01). Moreover, mothers of preterm babies were less likely to initiate breastfeeding than mothers who delivered on term with 92.0 and 95.9% initiation rates, respectively (*p* < 0.05). Overall, 1089/1822 (59.8%) initiated breastfeeding within 1 h, 28.6% (521/1822) initiated breastfeeding within 5 h, 4.6% (84/1822) within 10 h, 2.6% (47/1822) after 10 h and 4.4% did not initiate breastfeeding at all.

### Exclusive breastfeeding patterns

The exclusive breastfeeding rates were calculated based on the number of infants within a specific age group at the interview time who were exclusively breastfeeding out of the total number of infants within the same age group. The mean rate of exclusive breastfeeding among the 818 infants of 6 months or below was 44.3%, with rates decreasing by age. The rates of exclusive breastfeeding within the past 24 h at the first, third and sixth months of life were 50.0% (12/24), 47.1% (154/327) and 34.6% (27/78), respectively. As reported by the mothers, exclusive breastfeeding among infants of 6 months and above continued for 38/1004 (3.8%) infants, of whom 30 were 7–10 months and eight children were above 10 months of age.

**Fig. 2:**
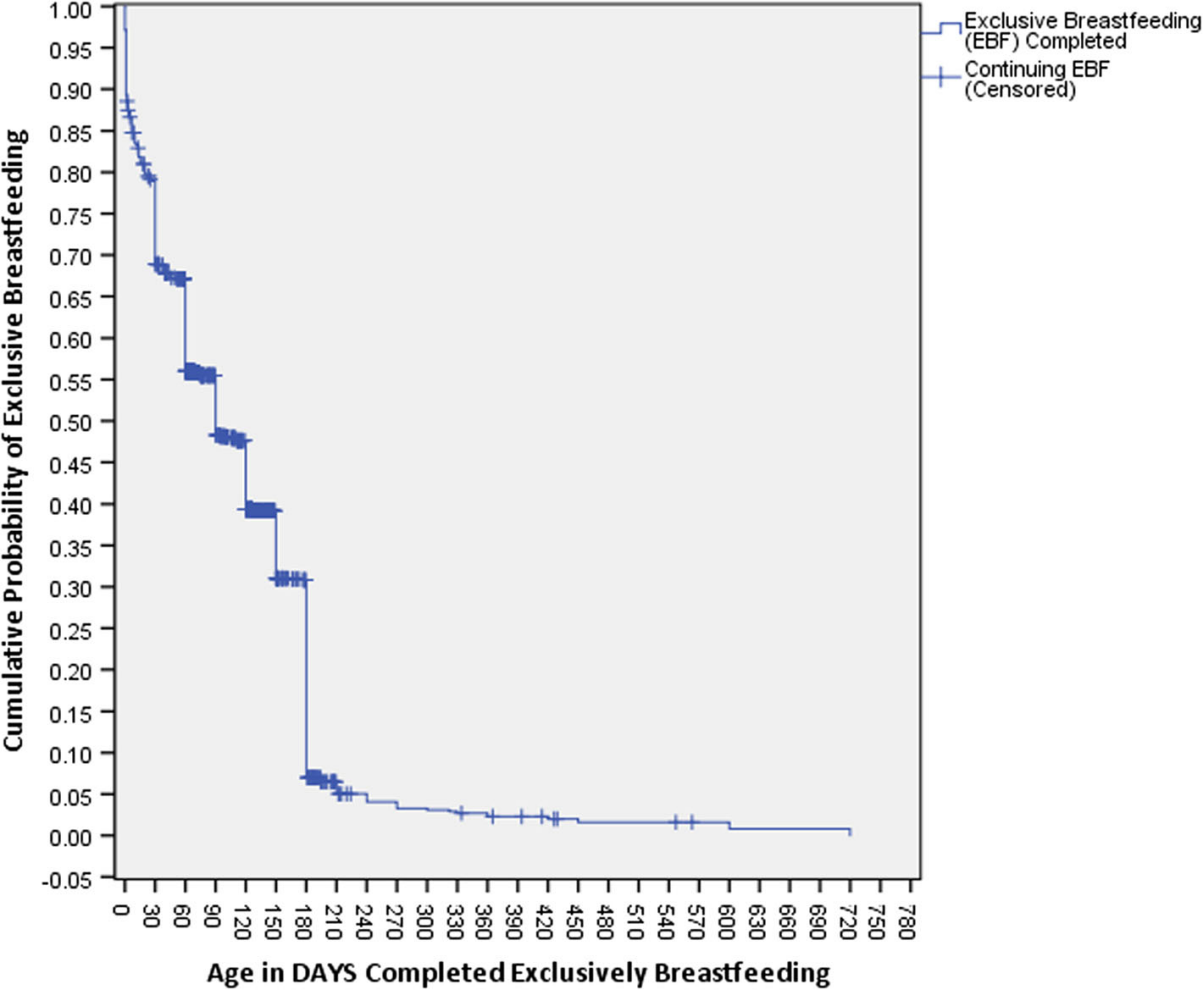
The survival analysis test curve reflecting the cumulative, exclusive breastfeeding experience among all children who had initiated breastfeeding in this study (*n* = 1741)

Figure 2 displays the survival analysis test curve which reflects the cumulative, exclusive breastfeeding (EBF) experience among all children who had initiated breastfeeding (*n* = 1741), including both those who had completed EBF and those who were continuing EBF at the time of interview. The mean and median duration of exclusive breastfeeding were 3.7 (95% CI 3.4,3.9) months and 3 months (95% CI 2.8,3.3), respectively. The median duration of any breastfeeding (not shown in the figure) was 12 months (95% CI 11.2,12.7).

### Overall feeding patterns by child’s age at interview

**Fig. 3:**
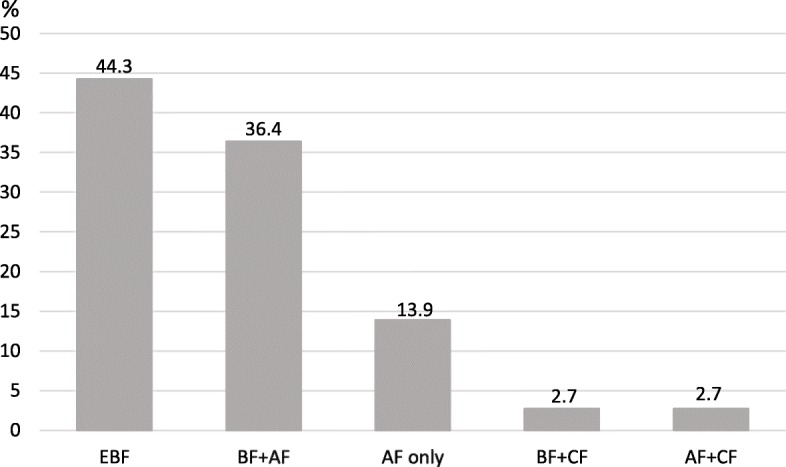
The overall feeding pattern of children < 6 months at the time of the interview (*n* = 818). *AF* Formula feeding, *EBF* Exclusive Breastfeeding, *BF* Breastfeeding, *CF* Complementary Feeding

**Fig. 4:**
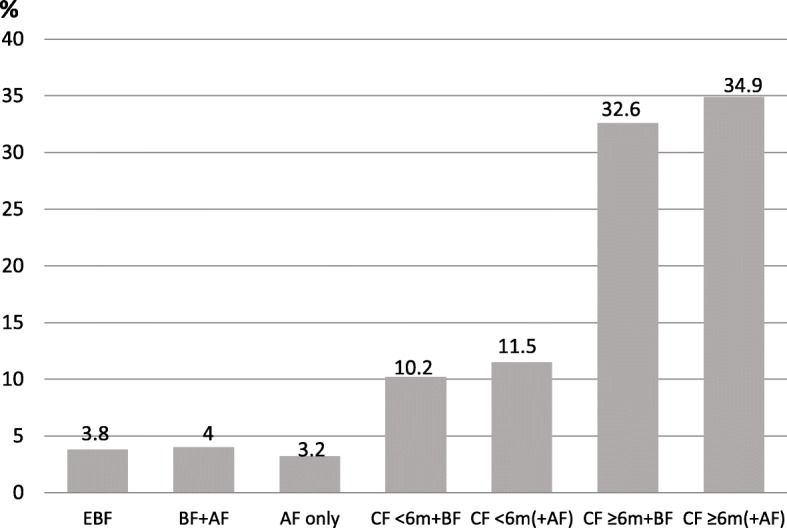
The overall feeding pattern of children ≥6 months at the time of the interview (*n* = 1004). *AF* Formula feeding, *EBF* Exclusive Breastfeeding, *BF* Breast Feeding, *CF* Complementary Feeding, CF<6m/≥6m indicate age (months) of child when CF was introduced

Figures 3 and 4 display the infants’ feeding patterns by age at the time of the interview. Among the 818 children below 6 months of age at the time of the interview the majority 682 (83.4%) received breastmilk to some extent including 362 (44.3%) who were exclusively breastfed and 320 (39.1%) who received a combination of breastmilk and formula milk or complementary feeding (Fig. 3). The remaining 136 children below 6 months of age (16.6%) received formula feeding only or in combination with complementary feeding (Fig. 3).

Among the 1004 older children, of 6 months of age or above at the time of the interview, the feeding patterns differed. As shown in Fig. 4, 506 of the children in this age group (50.4%) were breastfeeding to some extent. It further shows that most children, 89% (894/1004) were receiving complementary food in combination with breastmilk or infant formula. Among the children aged 6 months or above, a total of 21.7% (218/1004) had been introduced to complementary feeding early, i.e. before 6 months of age (Fig. 4). In total, regardless of age, 65.1% (1186/1822) of the studied children were breastfed to some extent at the time of the interview. Additionally, regardless of age, a total of 51.5% (938/1822) of the study sample received complementary feeding.

Table 3 summarizes the breastfeeding practices among the study participants in comparison to the WHO recommendations. While breastfeeding initiation and introduction of complementary feeding were considered “good” according to the WHO recommendations, breastfeeding (exclusive or any) and duration were considered fair or poor by WHO standards.

**Table 3:** Study findings compared to the WHO infant and young child feeding indicators

Practices defined by WHO^a^	Study findings % (*n*)	WHO rating^b^(Range for this rating in %)
Initiation of breastfeeding (% of babies breastfed within 1 h of birth).	59.8 (1089/1822)	Good (50–89)
Exclusive breastfeeding (% of babies 0 – < 6 months of age exclusively breastfed in the last 24 h).	44.3 (362/818)	Fair (12–49)
Duration of breastfeeding (median duration in months of breastfeeding of children under 3 years of age).^c^	12 (1741)	Poor (0-17)
Complementary feeding (% of breastfed babies 6 – < 10 or 7 – < 10 months of age who received complementary foods in the last 24 h).	79.9 (311/389)	Good (80–94)

^a^World Health Organization. Infant and young child feeding a tool for assessing national practices, policies and programmes. WHO. Geneva 2003

^b^The rating options are poor/fair/good/very good for all variables but the percentage varies depending on practise

^c^The children in this study were ≤ 2 years of age

## Discussion

In this cross-sectional study, most of the children (95.6%) were introduced to breastfeeding and as recommended, the majority initiated breastfeeding within an hour after delivery earning a rating of “good” according to the WHO indicators. Additionally the WHO indicator of exclusive breastreeding rate was considered “fair” and the duration of breastfeeding was considered “poor”. Most mothers whose babies were 6 months or older (89%) reported giving complementary food to their babies. However, early initiation of complementary feeding among infant being 6 months of older was reported among 21.7%.

In other countries breastfeeding initiation varies between 17.7 to 98.4% [30]. The initiation rate of breastfeeding in this study of 95.6% is high, and is on par with other studies conducted in the UAE, indicating that hospitals, regardless of being governmental or private and regardless of whether they have implemented the WHO and UNICEF Baby Friendly Initiative or not, are encouraging and supporting mothers to start breastfeeding [16, 17]. The finding of Emirati mothers initiating breastfeeding to a lower extent than the other nationality groups is somewhat surprising as the UAE is a Muslim country and Islam emphasizes the importance of breastfeeding. This indicates that there is a need to understand what socio-cultural factors and barriers are impacting mothers’ decisions on whether or not to initiate and continue breastfeeding to better support all women, regardless of nationality [18].

An early initiation of breastfeeding promotes exclusive breastfeeding by bonding between mother and child. This helps the mother breastfeed her child for a longer duration which increases the chances of successful breastfeeding, and generally lengthens the duration of breastfeeding [31, 32]. Early initiation of breastfeeding occurred in 59.8% of the infants, which, according to the WHO rating is considered “good” (range 50–89%) [13]. The results are lower than those reported by other researchers in the UAE (75–80%) [16, 17, 33]. In this study almost a third of the children were delivered through cesarean section, a practice that is becoming more and more common in the UAE [34]. This may delay the initiation of breastfeeding beyond an hour and could potentially explain why the current results are slightly lower than in previous studies. Other reasons may be due to variations in hospital practices and support to mothers immediately after delivery.

The exclusive breastfeeding during the last 24 h among children below 6 months of age at the time of the interview was 44.3%, which is rated “fair” according to the WHO tool, indicates that there is a need for improvements. The survival analysis, showing the cumulative exclusive breastfeeding experience among all children who initiated breastfeeding, showed that only 7% of all children who initiated breastfeeding in this study were exclusively breastfeeding at 6 months. Although the results in this study is not at the desired level, the overall rates of exclusive breastfeeding are slightly better than those reported in previous studies conducted in Abu Dhabi, Dubai and Al Ain where the rate of exclusive breastfeeding at 6 months varied between 0.0–1.9% [16, 17]. One of the other Emirates, Sharjah, has managed to improve the breastfeeding rates over the past years as a result of a targeted multisectorial and multidirectional breastfeeding campaign that has been ongoing since 2011. This campaign has provided professional support to get all healthcare facilities accredited as baby friendly, offering breastfeeding rooms at workplaces and in public places, and forbidding promotion of baby formula in nurseries and healthcare facilities as some of the main activities [15]. While it is not always easy to incorporate international campaigns in local settings, an exchange of experiences within the UAE would likely be beneficial as stakeholders could support each other to reach the goals set by the WHO of having 50% of infants being exclusively breastfed at 6 months of age in 2025 [35]. The WHO further recommends continued breastfeeding until 2 years of age, which was a common practice in the 1970’s in the UAE [36]. However, in the current study continued breastfeeding up to the age of two was very uncommon, again revealing the need to understand what factors are impacting infant feeding practices [12, 37].

In the study, most mothers who did not exclusively breastfeed their children for 6 months used formula feeding instead, a practice that is discouraged according to the recommendations. However, if mothers do not breastfeed, an age appropriate formula that meets the infants’ nutritional needs is still the preferred option over the early introduction of complementary feeding. Although there are slight variations in the recommendations of initiation of complementary feeding between countries there seem to be a common understanding that complementary feeding should not to be introduced before 4 months at the earliest, due to an immature gastrointestinal system [38]. Early introduction of complementary feeding results in an increased risk of the child having allergies and interferes with successful breastfeeding [39]. According to the WHO, timely complementary feeding means introducing semisolid food by 6 months of age whereas some countries like the United Kingdom means that complementary feeding can be initiated from 4 to 6 months of age if the child seems to be ready [40]. Previous studies in the Middle East have shown that complementary feeding is often introduced earlier than recommended [16, 41, 42]. In this study 21.7% of the children of 6 months or older had been introduced to complementary feeding before they were 6 months, a practice that has been associated with suboptimal health and growth in infants in other studies [43, 44]. In a recent study conducted by Radwan in the UAE, 83.5% of the children had received complementary feeding before 6 months of age [17]. The differences in findings may be related to study methodology or differences in the sample characteristics. The current study presents the result of a heterogeneous sample with around half of the mothers being non-UAE nationals whereas the study by Radwan only included Emirati mothers [17]. Furthermore, studies have shown that introducing complementary feeding later than 10 months has been associated with a higher risk of micronutrient deficiencies [41, 45]. In this study most children were receiving complementary feeding before 10 months of age which is good as breastmilk doesn’t offer sufficient vitamins and minerals at that age [9]. Among the few children who were introduced to complementary feeding later than 10 months, it would be of importance to investigate their health status.

The WHO rating tool is primarily designed for national needs assessments and hence the ratings in this study should be applied and interpreted with caution. Nevertheless, the ratings of “poor”, “fair”, “good” and borderline “fair to good” were obtained. These findings indicate that there is a need to design and implement effective strategies to improve the duration of exclusive breastfeeding, as the current practices and policies are not conducive to the successful meeting of the national and local goals to improve infant health and reduce childhood obesity [28, 46].

Since it is a cross-sectional study that included mothers of infants and young children up to 2 years of age, there may be some problems of recall on breastfeeding practices particularly among mothers with older children in the study and this can be seen as a limitation. Using the WHO indicator for exclusive breastfeeding rates among children being below 6 months of age in this study may have its limitations as many children were 0–3 months old, which may lead to an overestimation. The UAE is a multinational country where around 80% of the population in Abu Dhabi are expatriates [29]. Although the results in this study can not be seen as representative for all of the UAE, a major strength of the study is that infants, both UAE nationals and expatriates were recruited from the majority of the centers rendering maternal and health services located in various geographical areas in Abu Dhabi capital district as well as the community making the sample likely to be representative of Abu Dhabi.

## Conclusions

Although the breastfeeding initiation was considered “good” according to the WHO infant feeding indicators, the proportion of children being exclusively breastfed until 6 months was fair and the duration of exclusive breastfeeding was considered “poor”, indicating suboptimal infant feeding practices. These findings suggest the need for further research to understand potential barriers to breastfeeding as well as variations in feeding practices and their relation to sociodemographic factors in order to develop appropriate strategies to promote and support breastfeeding in Abu Dhabi. Another important step would be to enforce all healthcare facilities providing maternal and child health services and early childhood educational settings to get the global “Baby Friendly” accreditation by WHO and UNICEF.

## Abbreviations

SD: Standard Deviation
UAE: United Arab Emirated
UNICEF: United Nations Children’s Fund
WHO: World Health Organization
